# Normalization method for relative cerebral blood flow influences sex and cognitive status effects in nondemented older adults

**DOI:** 10.1007/s11682-026-01174-2

**Published:** 2026-07-01

**Authors:** Danielle L. Sanchez, Ilana J. Bennett

**Affiliations:** Department of Psychology, University of California, Riverside, Riverside, CA 92521 USA

**Keywords:** Pulsed arterial spin labeling, Cerebral blood flow, Gray matter, Older adults, Mild cognitive impairment

## Abstract

Cerebral blood flow (CBF) is lower in males than females in cortical gray matter, but these sex effects are unknown in the hippocampus. Potential interactions between sex and cognitive status on CBF, which may contribute to the greater risk for females to develop mild cognitive impairment (MCI), are also understudied. Moreover, these effects may vary when regional CBF is normalized relative to individual differences in reference region CBF (residual rCBF) compared to more common difference scores (traditional rCBF). The current study examined effects of sex, cognitive status, and their interaction on residual and traditional rCBF in the cortical lobes and hippocampus in 111 cognitively unimpaired older adults (CU; 61.3%) and 49 older adults diagnosed with MCI (46.9% female) from the Alzheimer’s Disease Neuroimaging Initiative (ADNI) with T1-weighted and perfusion-weighted magnetic resonance imaging data. As expected, rCBF was significantly lower in males than females in the occipital lobe, *p* = 0.010, but was unexpectedly significantly higher in males than females in the hippocampus, *p* < 0.001, with the sex effect being larger for traditional rCBF in the occipital lobe and residual rCBF in the hippocampus, *p*s < 0.024. Cognitive status significantly interacted with rCBF metric and region, *p*s < 0.014, but yielded no significant between group difference for any metric in any region, *p*s > 0.06. There were no significant interactions between sex and cognitive status, *p*s > 0.10. Taken together, these findings indicate that effects of sex on rCBF are region specific, independent of cognitive status, and vary with rCBF normalization method in non-demented older adults.

**Supplementary Information:**

The online version contains supplementary material available at https://doi.org/10.1007/s11682-026-01174-2.

## Introduction

Cerebral blood flow (CBF) dysfunction has garnered significant attention in aging and dementia research in recent years (Alisch et al., 2021; Sierra-Marcos, 2017). CBF is a metabolically demanding process that provides the brain with a constant supply of oxygen and nutrients to function properly (Wang et al., 2011). These nutrients are delivered through a complex system of cerebral arteries throughout the brain and, when disrupted, result in damage to the central nervous system and may induce accelerated aging (Iadecola, 2004; Van Beek et al., 2008). Autoregulatory processes in the brain are well-equipped to handle momentary or acute increases or decreases in blood flow in aging; however, chronic disruptions at either extreme (i.e., hypo- and hyper-perfusion) can result in deleterious effects and irreversible brain injury (Zlokovic, 2011). These chronic disruptions may explain reports of CBF differences (both lower and higher) in older adults diagnosed with mild cognitive impairment (MCI) who are at increased risk of Alzheimer’s disease relative to cognitively unimpaired (CU) older adults (Sierra-Marcos, 2017). However, potential interactions with sex remain untested despite consistent reports of lower CBF in males relative to females (Liu et al., 2012; Makwana et al., 2020) in addition to females having higher rates of MCI and worse vascular health (Barnes, 2017; Espeland et al., 2020; Haring et al., 2013; Kim et al., 2018; Ruitenberg et al., 2005). The “sex-focused” (versus “sex-controlled”) examination of CBF in nondemented older adults needed to address this gap may inform sex differences in the risk for developing MCI and Alzheimer’s disease.

CBF can be measured with arterial spin labeling (ASL), a non-invasive magnetic resonance imaging (MRI) technique that capitalizes on water molecules in arterial blood (Clement et al., 2022; Williams et al., 1992). In general, ASL works by subtracting labeled images, acquired after changing the magnetization of arterial blood water in the neck region and then waiting for this “magnetically labeled” blood water to diffuse the brain, from control images without magnetically labelling blood water, creating a perfusion-weighted image that is proportional to CBF (Alsop et al., 2015). There are two MRI sequences commonly used to acquire ASL data: *pseudocontinuous ASL (pCASL)* and *pulsed ASL (pASL)*. Although current recommendations are to use pCASL given its superior signal-to-noise ratio (Alsop et al., 2015), many large-scale, multi-site studies of Alzheimer’s disease have implemented pASL (Apostolova et al., 2021; McKay et al., 2023), including the Alzheimer’s Disease Neuroimaging Initiative (ADNI)(Weiner et al., 2017). There are also two classes of metrics used to measure CBF: *absolute CBF* and *relative CBF (rCBF)*. Absolute (or quantitative) CBF can be measured when separate calibration images are also acquired. rCBF is typically estimated by subtracting or dividing mean CBF in a reference region (e.g., whole-brain gray matter) from mean CBF in the region of interest (Aslan & Lu, 2010; Lacalle-Aurioles et al., 2013). However, this *traditional rCBF* approach assumes that there are minimal individual- or group-level differences in the magnitude of CBF in the reference region, which may not be the case if there is global hypo- or hyper-perfusion between groups of interest. An alternative *residual rCBF* approach that normalizes regional CBF relative to reference CBF may better account for global differences in perfusion between groups, as has been shown for regional volume (Jack et al., 1989), but it has not yet been applied to CBF.

Prior ASL studies have consistently found that CBF is lower in males than females in cortical regions. This sex group difference has been reported when using absolute CBF from pASL (Liu et al., 2012; Makwana et al., 2020) and pCASL (Alisch et al., 2021; Leidhin et al., 2021; Liu et al., 2016), indicating that it is independent of ASL sequence. Notably, none of these studies examined sex differences in CBF in the hippocampus, a region known for being particularly vulnerable in Alzheimer’s disease.

ASL studies have yielded mixed effects of cognitive status when comparing MCI and CU older adults (Sierra-Marcos, 2017). When using pASL, lower CBF in MCI than CU older adults has been reported in parietal, temporal, and occipital regions in some studies (Alexopoulos et al., 2012; Camargo & Wang, 2023; Johnson et al., 2007), yet others report no significant effects of cognitive status on CBF (Mattsson et al., 2014; Riederer et al., 2018). In contrast, these pASL studies consistently report no significant differences between MCI than CU older adults for CBF in the hippocampus. Importantly, they all statistically controlled for sex when examining cognitive status effects on CBF.

The extent to which sex differences in CBF interact with cognitive status remains unknown. One pCASL study found lower absolute CBF in older adults diagnosed with MCI relative to older adults with subjective cognitive complaints in cortical (parietal), but not hippocampal, gray matter (Binnewijzend et al., 2013). Sex effects within each cognitive status group further revealed lower CBF in males relative to females in occipital and parietal regions for older adults with subjective cognitive complaints, but no sex differences in CBF for the MCI group in any region. Although these findings are suggestive of an interaction between sex and cognitive status, they did not directly test for it. Moreover, they may have underestimated effects of cognitive status as their CU group consisted of older adults with subjective cognitive complaints who are further along the Alzheimer’s disease continuum.

The current study sought to address gaps in prior work by examining effects of sex (male, female) and cognitive status (CU, MCI) on cortical lobar (frontal, parietal, temporal, occipital) and hippocampal rCBF using both traditional rCBF and the novel residual rCBF measure from pASL data in non-demented older adults from the ADNI. We first tested for global hypo- or hyper-perfusion between groups by examining effects of sex and cognitive status on mean CBF in the reference region. We then examined relationships between mean CBF in the reference region and both traditional and residual rCBF in each region of interest, predicting that these relationships would be attenuated for residual rCBF if it better accounted for global differences in perfusion between groups. Finally, we assessed effects of sex, cognitive status, and their interaction on rCBF and whether these effects differed when using residual or traditional rCBF. We expected to replicate known effects of sex on cortical lobar rCBF (lower in males than females), with no clear predictions for the hippocampus given the absence of prior pASL studies. We further expected significant effects of cognitive status on cortical lobar, but not hippocampal, rCBF, but were agnostic to the direction of the effect given the mixed literature. If significant, we expected an interaction to show that the sex effect on cortical rCBF is larger in the CU than MCI group. Finally, we predicted that effects of sex and cognitive status would differ when using traditional versus residual rCBF metrics, with the former being an artifact of unaccounted global differences in perfusion between groups.

## Methods

### The alzheimer’s disease neuroimaging initiative

Data used in the preparation of this article were obtained from the ADNI database (adni.loni.usc.edu). The ADNI was launched in 2003 as a public-private partnership, led by Principal Investigator Michael W. Weiner, MD. The primary goal of ADNI has been to test whether serial MRI, positron emission tomography (PET), other biological markers, and clinical and neuropsychological assessment can be combined to measure the progression of MCI and early Alzheimer’s disease. In the third phase (ADNI3; 2016–2022), ASL was acquired in all participants. Institutional Review Board approval and written informed consent were obtained from all participants or authorized representatives at each ADNI site.

### Participants

Data from 187 older adults in ADNI3 who had T1-weighted and perfusion-weighted MRI data within 12 months of their baseline visit were obtained from the ADNI3 database between August 15th, 2021, and September 9th, 2022. Of these participants, 17 cognitively unimpaired (CU) older adults and 10 older adults diagnosed with MCI had MRI scan parameters that differed from those described in the MRI Data Acquisition section and were excluded. Participant characteristics for the final sample of 160 older adults (68 CU female, 43 CU male, 23 MCI female, 26 MCI male) are presented in Table 1.

**Table 1:** Demographic data

	CU		MCI	
	Female	Male	Female	Male
N	68	43	23	26
Sex (%)	61.3%	38.7%	46.9%	53.1%
Age (M ± SD)	67.9 ± 7.7	71.2 ± 8.2	68.8 ± 7.9	75.4 ± 8.4
Education (M ± SD)	16.2 ± 2.3	17.1 ± 2.5	15.7 ± 2.2	16.2 ± 2.4

Demographic data is shown separately for each cognitive status (cognitively unimpaired, CU; mild cognitively impaired, MCI) and sex (female, male) group. M ± SD = mean ± standard deviation

ADNI3 participants were characterized as CU using the following criteria: (1) a Clinical Dementia Rating (CDR) score of 0, (2) a Mini-Mental State Exam (MMSE) score between 24 and 30, (3) normal memory functioning adjusted for education as measured by the Logical Memory II (LM II) subscale from the Wechsler Memory Scale-Revised (WMS-R), (4) with or without subjective memory complaints made by the participant and verified by their study partner, (5) no significant impairment in activities of daily living (ADL), and (6) current medication stability for at least four weeks.

ADNI3 participants were characterized as having MCI using the following criteria: (1) a CDR score of 0.5, (2) MMSE score between 24 and 30, (3) abnormal memory function as determined by cut-off scores for the WMSR LM II, (4) express a subjective memory concern verified by a study partner or clinician, (5) some impairment in ADL where a diagnosis of AD cannot be made, and (6) medication stability for at least four weeks (with cholinesterase inhibitors and memantine stable for 12 weeks).

### MRI data

#### Acquisition

MRI data were acquired on 3 T Siemens Prisma scanners. A T1-weighted accelerated magnetization prepared rapid acquisition gradient echo (MP-RAGE) sequence was acquired with the following parameters: repetition time (TR) = 2300 ms, echo time (TE) = 2.98 ms, inversion time (TI) = 900 ms, flip angle = 9 degrees, 208 sagittal slices, field of view (FOV) = 256 mm, and spatial resolution = 1.0 × 1.0 × 1.0 mm.

A 3D proton-density weighted pASL sequence with 10 interleaved control and tag image pairs was acquired with the following parameters: TR = 4000 ms, TE = 20.26 ms, TI = 2000 ms, flip angle = 180 degrees, FOV = 240 mm, and spatial resolution = 1.9 × 1.9 × 4.5 mm. For each of the 20 volumes, axial slices were acquired in ascending interleaved order. The bolus duration (800 ms) was defined using a QUIPS II with thin-slice TI1 periodic saturation sequence (“Q2TIPS”) with echo-planar imaging (Luh et al., 1999).

#### Preprocessing

For each participant, raw T1-weighted and 3D pASL data were converted from DICOM to NIFTI format using dcm2nii (www.nitrc.org). FSL (FMRIB’s Software Library, www.fmri.ox.ac.uk/fsl) was used for all subsequent pre- and post-processing procedures.

Raw T1-weighted data underwent skull-stripping and brain masking using FSL’s brain extraction tool (BET; Smith, 2002), with the fractional intensity threshold set to 0.2 and the option for bias-reduction from residual non-brain voxels. Tissue type was segmented into gray matter, white matter, and cerebral spinal fluid using FMRIB’s Automated Segmentation Tool (FAST; Zhang et al., 2001).

Raw pASL data underwent motion correction using FMRIB’s Linear Registration Tool (MCFLIRT; Jenkinson et al., 2002) to align all volumes to the first volume. FSL’s Bayesian Inference for Arterial Spin Labeling (BASIL; Chappell et al., 2009, 2011) was then used to quantify CBF. A perfusion-weighted difference image was calculated as the average of pairwise subtractions of the motion-corrected control-tag repeats using the *asl_file* command. This difference image was input to the *oxford_asl* command using the option to conform the analysis to White Paper mode for partial volume corrections, which included specifying FAST-segmented gray matter and white matter volume estimates (Alsop et al., 2015). The analysis was limited to brain space by using a brain mask that was aligned from T1 space to a single volume average of the motion-corrected pASL control images with a linear transformation and 6 degrees of freedom. Additional acquisition options specified the inversion time, bolus duration, structural data (whole brain and brain-extracted) and defaulted to turned off arterial suppression (Chappell et al., 2011). Output from *oxford_asl* included a voxel-wise image of raw CBF in T1 space.

#### Regions of interest

Bilateral cortical lobar (frontal, parietal, temporal, and occipital) masks were extracted from FSL’s Montreal Neurological Institute (MNI) atlas (MNI-maxprob-thr0-1 mm) and binarized. For each participant, these lobar masks were aligned to their T1-weighted image using linear and nonlinear transformations and then multiplied by their FAST-segmented gray matter mask.

The left and right hippocampus was segmented from each participant’s T1-weighted image using the *run_first_all* command (FMRIB’s Integrated Registration and Segmentation Tool, FIRST; Patenaude et al., 2011), with the option for a 3-stage affine registration. A bilateral hippocampus mask was created by summing the segmentations across hemispheres.

A whole-brain gray matter reference mask was created for each participant by combining the T1-aligned, FAST-segmented bilateral cortical lobar and hippocampal masks.

#### rCBF calculations

For each participant, mean CBF values were calculated in each region of interest by multiplying each regional mask by their voxel-wise perfusion map in T1 space and averaging values across voxels. Traditional rCBF values were calculated for each bilateral cortical lobar and hippocampal region of interest by subtracting mean CBF in the whole-brain gray matter reference mask from mean CBF in each region.

Residual rCBF values were calculated for each bilateral cortical lobar and hippocampal region of interest using a residual normalization method that accounts for individual differences in mean CBF in the reference region (Jack et al., 1989). Group-level mean CBF in the whole-brain gray matter reference region and a group-level slope of the relationship between mean CBF in each region and in the reference region (β_region_) were calculated within the CU group to estimate these metrics in the absence of MCI-related CBF dysfunction. Residual rCBF values were then calculated separately for each participant in each region of interest using the equation: (participant mean CBF in a given region) − β_region_ ((participant mean CBF in the reference region) – (group-level mean CBF in the reference region). Sample calculations of traditional rCBF and residual rCBF are provided in Supplementary Table 1.

### Statistical analyses

Differences in the sample distribution between Sex (female, male) and Cognitive Status (CU, MCI) groups were assessed with a chi-square test of independence. Group differences in age and education were assessed using separate Sex × Cognitive Status univariate analyses of variance (ANOVA).

Correlations were conducted between rCBF in each region of interest and mean CBF in the reference region, separately for each rCBF metric (traditional, residual).

Group differences in mean CBF in the reference region were assessed with a Sex (male, female) × Cognitive Status (CU, MCI) univariate analysis of covariance (ANCOVA) that controlled for age. Group differences in regional rCBF were assessed with a Metric (traditional, residual) × Sex (male, female) × Cognitive Status (CU, MCI) × Region (frontal, parietal, temporal, occipital, hippocampal) mixed factorial ANCOVA that controlled for age. For significant effects, mean group averages or group differences (M_diff_) are reported ± standard error.

## Results

### Sample distribution and demographic effects

A chi-square test of independence revealed no significant differences in the sample distribution across Sex and Cognitive Status groups, *p* = 0.092. The Sex × Cognitive Status ANOVA for age revealed a significant main effect of Sex, *F*(1,156) = 12.72, *p* < 0.001, such that females were significantly younger (68.36 ± 0.96) than males (73.29 ± 0.99). No other effects for age or education attained significance, *p*’s > 0.07.

### Effects of sex and cognitive status on reference CBF

The Sex × Cognitive Status univariate ANCOVA that controlled for age for mean CBF in the whole-brain gray matter reference region revealed a significant main effect of Sex, *F*(1, 155) = 10.4, *p* = 0.002, η^2^ = 0.06, with lower CBF in males (62.74 ± 4.09) than females (81.21 ± 3.89). A significant main effect of Cognitive Status, *F*(1, 155) = 6.2, *p* = 0.014, η^2^ = 0.04, revealed lower CBF in the MCI (65.05 ± 4.60) than CU (78.90 ± 3.11) group.

### Correlations between reference CBF and regional rCBF

**Fig. 1:**
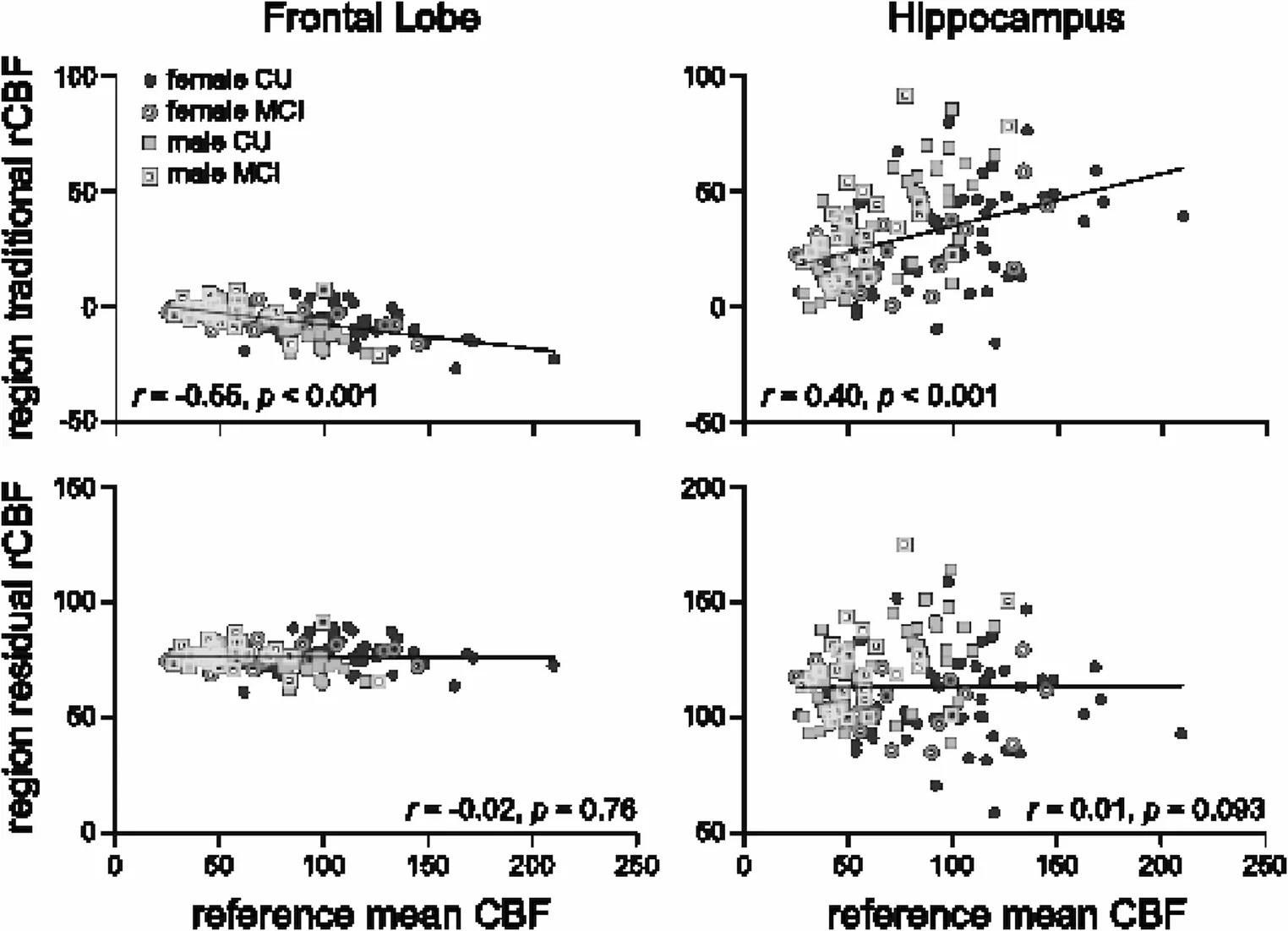
Scatterplots show relationships between mean CBF in the whole-brain gray matter reference region and both traditional rCBF (top) and residual rCBF (bottom) in the frontal lobe (left) and hippocampus (right). Data points are coded by sex (female, male) and cognitive status (cognitively unimpaired, CU; mild cognitive impairment, MCI) group

For the traditional metric (see Fig. 1, top), correlations between mean CBF in the whole-brain gray matter reference region and rCBF in each region of interest revealed a significant negative relationship for the frontal lobe, *r* = −0.55, *p* < 0.001; positive relationships for the parietal lobe, *r* = 0.33, *p* < 0.001, occipital lobe, *r* = 0.19, *p* = 0.019, and hippocampus, *r* = 0.40, *p* < 0.001; and were not significant for the temporal lobe, *p* = 0.17.

For the residual metric (see Fig. 1, bottom), these correlations revealed no significant relationships for any region, *p*s > 0.70.

### Effects of sex, cognitive status, and metric on regional rCBF

**Fig. 2:**
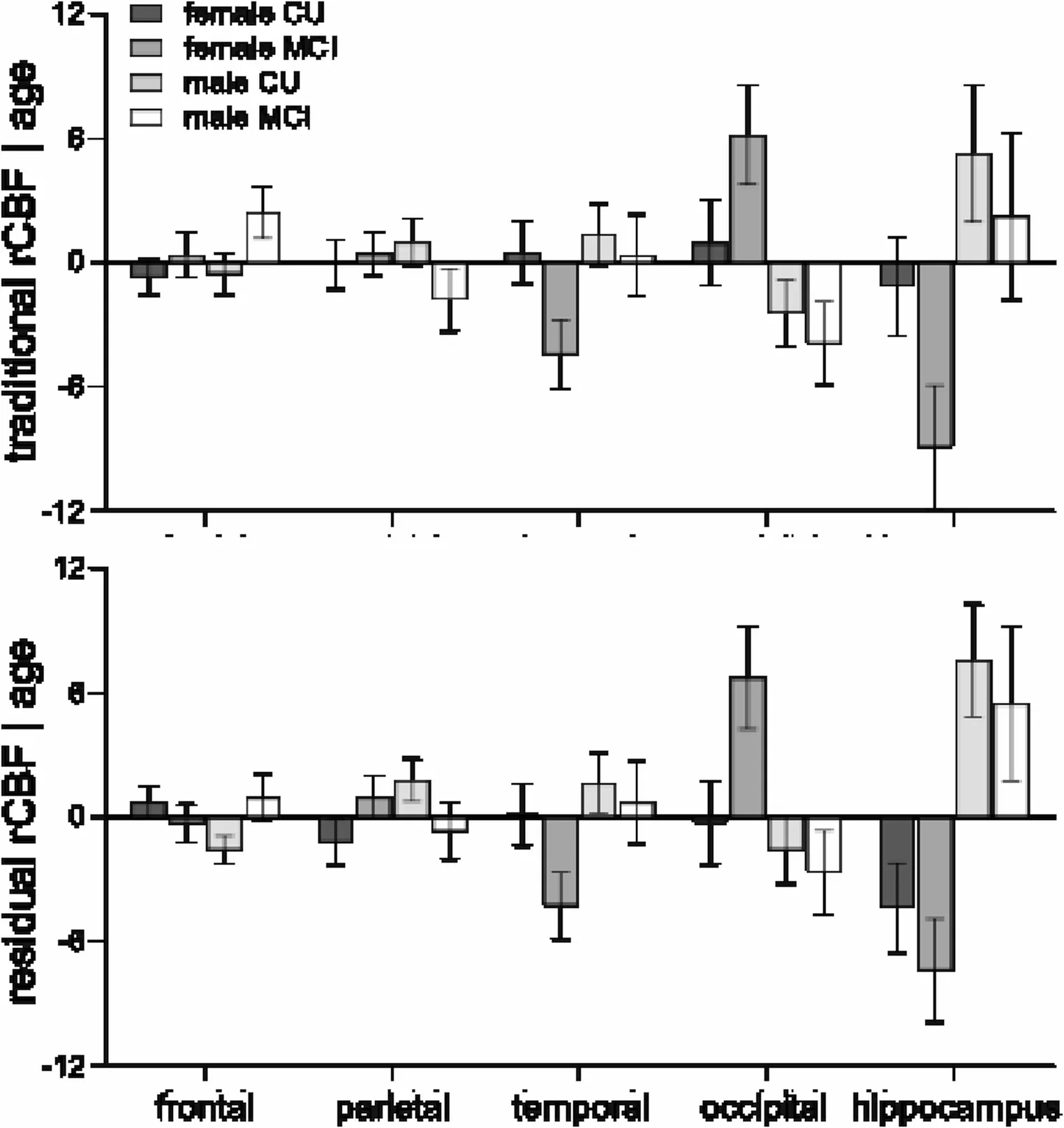
Separate bar graphs (mean ± standard error) for each rCBF metric (traditional, residual) show values for each sex (female, male) and cognitive status (CU, MCI) group in each cortical lobar and hippocampal region of interest when controlling for age

Results of the Metric × Sex × Cognitive Status × Region mixed factorial ANCOVA that controlled for age are presented in Fig. 2; Table 2. Results when also controlling for ADNI site are presented in Supplementary Table 2.

**Table 2:** rCBF descriptive statistics

Traditional rCBF	Female	Male	Sex F	CU	MCI	Cognitive Status F
Frontal	−5.5 ± 0.8	−4.2 ± 0.8	1.2	−5.9 ± 0.6	−3.8 ± 0.9	3.5
Parietal	2.4 ± 1.0	1.7 ± 1.1	0.2	2.6 ± 0.8	1.4 ± 1.2	0.7
Temporal	7.6 ± 1.3	10.6 ± 1.4	2.3	10.6 ± 1.0	7.6 ± 1.6	2.4
Occipital	−3.5 ± 1.7	−10.7 ± 1.8	**8.5**	−7.9 ± 1.3	−6.3 ± 2.0	0.5
Hippocampus	24.6 ± 2.4	33.9 ± 2.5	**6.8**	31.8 ± 1.9	26.7 ± 2.8	2.2
*Residual rCBF*						
Frontal	76.7 ± 0.7	76.1 ± 0.7	0.3	76.0 ± 0.5	76.7 ± 0.8	0.6
Parietal	84.7 ± 0.9	85.5 ± 1.0	0.3	85.2 ± 0.8	85.1 ± 1.1	< 0.1
Temporal	89.9 ± 1.3	93.3 ± 1.4	3.1	92.9 ± 1.0	90.3 ± 1.6	1.9
Occipital	78.9 ± 1.7	73.2 ± 1.8	**5.2**	74.6 ± 1.3	77.4 ± 2.0	1.4
Hippocampus	107.2 ± 2.1	120.6 ± 2.2	**18.5**	114.9 ± 1.7	112.8 ± 2.4	0.5

rCBF values (mean ± standard error) are provided separately for each sex and cognitive status group for each rCBF metric (traditional, residual) in each cortical lobar and hippocampal region of interest. Significant between-group *F*-test comparisons at *p* < 0.05 are bolded

Significant effects of Sex, *F*(1, 155) = 5.9, *p* = 0.016, η^2^ = 0.04, and Sex × Region, *F*(4, 620) = 7.8, *p* = 0.001, η^2^ = 0.05, revealed lower rCBF in males than females in the occipital lobe (M_diff_: −6.43 ± 2.47), *p* = 0.010, but higher rCBF in males than females in the hippocampus (M_diff_: 11.33 ± 3.28), *p* < 0.001. Significant effects of Metric, *F*(1, 155) = 3262.5, *p* < 0.001, η^2^ = 0.96, Metric × Sex, *F*(1, 155) = 10.4, *p* = 0.002, η^2^ = 0.06, and Metric × Sex × Region, *F*(4, 620) = 10.4, *p* < 0.001, η^2^ = 0.06, revealed that the sex effect in the occipital lobe was larger for the traditional (M_diff_: −7.21 ± 2.48), *p* = 0.004, than residual (M_diff_: −5.65 ± 2.48), *p* = 0.024, rCBF metric, whereas the sex effects in the hippocampus was larger for the residual (M_diff_: 13.40 ± 3.11), *p* < 0.001, than traditional (M_diff_: 9.27 ± 3.55), *p* = 0.010, rCBF metric. No other Sex group differences attained significance for other combinations of Metric or Region, *p*s > 0.08.

Significant effects of Metric × Cognitive Status, *F*(1, 155) = 6.2, *p* = 0.014, η^2^ = 0.04, and Metric × Cognitive Status × Region, *F*(4, 620) = 6.2, *p* < 0.001, η^2^ = 0.04, revealed no significant Cognitive Status group difference for any combination of Metric or Region, *p*s > 0.06. The only effect to approach significance was a trend for higher traditional rCBF in the MCI than CU group in the frontal lobe (M_diff_: 2.13 ± 1.13), *p* = 0.062.

No interactions with Sex and Cognitive Status attained significance, *p*s > 0.10.

## Discussion

The present study examined effects of sex, cognitive status, and their interaction on cortical lobar and hippocampal rCBF in non-demented older adults using ADNI3 pASL data and whether these effects differed when using traditional or residual rCBF metrics. Our results revealed four main findings. First, we observed significant group differences in sex and cognitive status in mean CBF in the reference region and that relationships between mean reference CBF and regional rCBF were attenuated when using residual rCBF compared to traditional rCBF, indicating that it better accounted for group differences global perfusion. Second, we replicated prior work showing lower rCBF in males than females in cortical gray matter (occipital lobes) and, for the first time, report a significant albeit opposite effect of higher rCBF in males than females in the hippocampus, with the sex effect in the occipital lobe being larger for traditional rCBF and the sex effects in the hippocampus being larger for the residual rCBF metric. Third, we found that cognitive status significantly interacted with rCBF metric and region, but yielded no significant between group difference for any metric in any region, replicating prior work showing no effect of cognitive status on hippocampal rCBF. Fourth, we observed no significant interaction between sex and cognitive status on rCBF in any regions of interest, suggesting that these variables have independent effects on cerebral blood flow.

Mean CBF in the whole-brain gray matter reference region was significantly lower in males than females and in the MCI than CU group. This demonstrates a violation of the common assumption that CBF in the reference region is constant across groups of interest. One implication is that traditional rCBF metrics that simply take the differences (or ratio) between CBF in a reference region and CBF in a region of interest can scale with these group differences, as individuals or groups with global hyper-perfusion (e.g., higher CBF in the reference region for females and CU older adults) have the potential for larger traditional rCBF values. This is seen by the significant negative correlations observed between mean CBF in the whole-brain gray matter reference region and traditional rCBF in each region of interest. Regional group differences may therefore be an artifact of this relationship when using traditional rCBF. Importantly, these relationships were not significant for residual rCBF in any region of interest, indicating that the residual method that normalized regional CBF relative to the magnitude of CBF in each individuals’ gray matter reference region is a more optimal approach for detrending global group differences in reference CBF. As such, we recommend residual rCBF over traditional rCBF for future pASL studies.

We replicated a significant effect of sex for cortical rCBF and observed a novel, opposite effect of sex for hippocampus rCBF that were independent of cognitive status (and age). Males had lower rCBF in the occipital lobe than females, in line with results from numerous prior pASL (Liu et al., 2012; Makwana et al., 2020), CASL (Parkes et al., 2004), and pCASL (Alisch et al., 2021; Leidhin et al., 2021; cf., Liu et al., 2016) studies that used absolute CBF, providing confidence that our rCBF metrics accurately capture the direction of the sex difference. In contrast, males had higher rCBF in the hippocampus relative to females. Although we did not have a specific prediction for the sex effect in the hippocampus given the absence of prior pASL work, we were surprised that the direction was opposite to that seen in the occipital lobe. One possibility is that these regional differences in rCBF may be driven by sex differences in hippocampal volume, as females display lower hippocampal volume relative to males (Raz et al., 2004; cf. Tan et al., 2016) and smaller volumes have been linked to lower rCBF (Alsop et al., 2008; Petersen et al., 2022). However, the direction of the sex difference in rCBF in the hippocampus remained unchanged when controlling for normalized hippocampal volume (see Supplementary Material). Thus, additional research is needed to replicate the observed sex differences in hippocampal blood flow and explore alternative interpretations.

Of note, the magnitude of the sex effect differed by rCBF metric, with larger sex group differences in the occipital lobe for traditional rCBF (lower in males than females) and in the hippocampus for residual rCBF (higher in males than females). This provides some confidence that the overall pattern of sex group differences remains stable across rCBF metrics. However, as detailed above, it does show that results can be overestimated (occipital lobe) or underestimated (hippocampus) when individual and group differences in reference region mean CBF are not fully accounted for (traditional rCBF) relative to when they are (residual rCBF). For example, global hyper-perfusion in one group would present as higher mean CBF in the reference region and yield what appear to be larger traditional rCBF values.

Although we found significant Metric × Cognitive Status and Metric × Cognitive Status × Region interactions, there was no significant main effect of cognitive status on cortical lobar or hippocampal rCBF and no significant cognitive status group difference for any combination of metric or region. The only effect to approach significance was a trend for higher traditional rCBF in the MCI than CU group in the frontal lobe, which is the opposite direction of significant cognitive status group differences reported in prior pASL work (Alexopoulos et al., 2012; Camargo & Wang, 2023; Johnson et al., 2007), but consistent with reports of no significant effects of cognitive status on cortical CBF (Mattsson et al., 2014; Riederer et al., 2018). The non-significant hippocampal result also replicats prior pASL studies (Johnson et al., 2007; Alexopoulos et al., 2012; Mattsson et al., 2014; Riederer et al., 2018; Camargo & Wang, 2023). Together, these pASL findings indicate that differences in blood flow between MCI and CU older adults are small and variable across regions and samples. Because CBF differences between MCI and CU older adults have been more consistently reported when using pCASL sequences (Camargo & Wang, 2023; Dolui et al., 2017, 2020; Soman et al., 2021), we recommend pCASL over pASL for future studies interested in cognitive status effects.

Finally, we found no evidence of an interaction between sex and cognitive status on rCBF in any of our regions of interest. Results from one prior pCASL study were suggestive of an interaction between cognitive status and sex on rCBF in cortical gray matter, but not hippocampus (Binnewijzend et al., 2013), although it was not directly tested. Prior longitudinal pCASL work also found that sex interacted with a different marker of Alzheimer’s disease risk (APOE ε4) when examining age-related changes in rCBF such that female APOE ε4 carriers had the largest age-related decline in both cortical and hippocampal blood flow relative to male APOE ε4 carriers, whereas non-carriers had no sex differences in rCBF changes with age (Wang et al., 2021). Of note, the patterns of sex and cognitive status effects observed here were attenuated but otherwise unchanged when controlling for APOE ε4 status in a subset of participants (Supplementary Material). Future studies should explore additional measures that can affect rCBF and may interact with cognitive status and/or sex, such as menopause and hormone therapy, both of which influence ovarian hormone levels. Estrogen, in particular, is thought to have a neuroprotective role on cerebrovascular functioning (Birge, 1996), with lower CBF in males than females, as was observed here, being attributed to elevated estrogen levels in women (Alisch et al., 2021), and lower CBF in post- versus pre-menopausal females attributed to its reduction following menopause (Liu et al., 2016).

Limitations of the current study include our decision to use the Siemens pASL sequence given its wider implementation across ADNI sites and therefore larger sample size (*n* = 123) relative to the pCASL sequence (*n* = 70), likely due to its shorter scan times that has made it easy to implement in both large- and small-scale aging and dementia research studies (Alsop et al., 2015; Apostolova et al., 2021; McKay et al., 2023; Weiner et al., 2017). However, pASL suffers from reduced labeling efficiency and a lower signal-to-noise ratio that may introduce additional artifacts that could obscure effects of interest (Alsop et al., 2015; Wang, 2014; Wu et al., 2009), such as the cognitive status effects of interest here. Procedural issues with data collection in ADNI3 (e.g., no calibration image) also limited us to calculate rCBF (i.e., higher or lower than a given reference region), but not absolute CBF. This created an opportunity for us to evaluate different normalization methods, finding that, whereas traditional rCBF may be influenced by individual and group differences in blood flow in the reference region, these artifacts are mitigated by the residual rCBF metric. ADNI data is also obtained from predominately “WEIRD” (White, educated, industrialized, rich, and democratic) and relatively healthy (e.g., free of serious cardiovascular health related issues) older adult sample compared to the larger national population, limiting the generalizability of our findings and introducing potential confounds such as cohort and survival effects. Lastly, after dividing the sample by sex and cognitive status, subgroups became relatively small (e.g., 23 MCI females), which may have limited our statistical power to detect true differences within our sample.

## Conclusion

In summary, the current pASL paper revealed significant effects of sex and cognitive status on mean CBF in a commonly used gray matter reference region and that the novel application of a residual rCBF metric that normalizes regional CBF relative to individual differences in reference CBF better accounted for these group differences than a traditional rCBF difference score. Both rCBF metrics replicated prior findings of lower rCBF in males than females in the occipital lobe and extended this literature to show, for the first time, higher rCBF in males than females in the hippocampus. Importantly, the traditional rCBF metric overestimated the magnitude of these sex effects in the occipital lobe and underestimated them in the hippocampus, likely an artifact of having not fully accounted for the sex group difference in mean reference CBF compared to the residual rCBF metric. We further found no significant effect of cognitive status for any rCBF metric in any region of interest. Finally, we did not find any evidence that sex and cognitive status have interactive effects on rCBF in any region, supporting the notion that these variables have independent effects on CBF. The robust sex effects observed here, coupled with the non-significant cognitive status effects, highlight the need for a sex-focused (instead of sex-controlled) approach to examinations of cerebrovascular health in nondemented older adults at risk for Alzheimer’s disease.

## Supplementary Information

Below is the link to the electronic supplementary material.

Supplementary Material 1 (DOCX 31.0 KB)

## Data Availability

No datasets were generated or analysed during the current study.
