# Supplementary material for: Normalization method for relative cerebral blood flow influences sex and cognitive status effects in nondemented older adults
Source: Brain Imaging Behav. 2026 Jul 1;20(4):104. doi: 10.1007/s11682-026-01174-2 (PMC13323295; doi:10.1007/s11682-026-01174-2)
Supplement: Supplementary file 1 — Supplementary Material 1 (DOCX 31.0 KB) [file 11682_2026_1174_MOESM1_ESM.docx]

**Supplementary Material**

**Supplementary Table 1.** Examples of rCBF calculations

| Participants | Mean CBF  in Reference | Mean CBF  in Region | Traditional rCBF in Region | Residual rCBF in Region |
| --- | --- | --- | --- | --- |
| CU 1 | 53.9 | 54.5 | 0.6 | 97.5 |
| CU 2 | 111.5 | 115.9 | 4.4 | 110.0 |
| CU 3 | 148.2 | 132.5 | -15.7 | 95.5 |
| MCI 1 | 99.0 | 80.2 | -18.8 | 84.9 |
| MCI 2 | 58.1 | 56.6 | -1.5 | 96.0 |
| MCI 3 | 40.8 | 37.8 | -3.0 | 91.9 |

*Note.* Sample data mean cerebral blood flow (CBF) data in a reference region and a region of interest are provided for three cognitively unimpaired older adults (CU) and three individuals diagnosed with mild cognitive impairment (MCI). Traditional relative CBF (rCBF) values are calculated for each participant in the region of interest by subtracting their mean CBF in the reference from their mean CBF in the region. Residual rCBF values are calculated for each participant in the region of interest by subtracting their mean CBF in the region from the CU group’s average mean CBF in the reference region (104.5 in this example), multiplying that value by the CU group’s slope of the relationship between mean CBF in the region and the reference (0.85 in this example), and subtracting that value from the participant’s mean CBF in the region.

**Supplementary Table 2.** ANCOVAs results when accounting for ADNI site.

|  | Not Controlling for ADNI Site | Controlling  for ADNI Site |
| --- | --- | --- |
| Sex | **5.9 (0.016)** | 3.3 (0.070) |
| Cognitive Status | 1.0 (0.324) | 0.4 (0.542) |
| Sex × Cognitive Status | 0.1 (0.790) | 0.5 (0.479) |
| Metric | **3262.5 (<0.001)** | **2552.3 (<0.001)** |
| Metric × Sex | **10.4 (0.002)** | **19.8 (<0.001)** |
| Metric × Cognitive Status | **6.2 (0.014)** | 1.4 (0.247) |
| Metric × Sex × Cognitive Status | 1.9 (0.168) | 0.3 (0.856) |
| Region | **2.2 (0.066)** | **2.5 (0.045)** |
| Region × Sex | **7.8 (<0.001)** | **5.7 (<0.001)** |
| Region × Cognitive Status | 1.3 (0.275) | 0.3 (0.905) |
| Region × Sex × Cognitive Status | 1.3 (0.253) | 0.3 (0.858) |
| Metric × Region | 0.9 (0.451) | 1.4 (0.221) |
| Metric × Region × Sex | **10.4 (<0.001)** | **19.8 (<0.001)** |
| Metric × Region × Cognitive Status | **6.2 (<0.001)** | 1.4 (0.250) |
| Metric × Region × Sex × Cognitive Status | 1.9 (0.106) | <0.1 (0.998) |

*Notes. F*-statistics (*p*-values) are provided for the Metric (traditional, residual) × Sex (male, female) × Cognitive Status (CU, MCI) × Region (frontal, parietal, temporal, occipital, hippocampal) mixed factorial analysis of covariance (ANCOVA) that either controlled for age (left) or age and the 17 ADNI sites (right; included as separate predictors in the model). Significant effects at *p* < 0.05 are bolded. Finding that effects with Cognitive Status (the Metric × Cognitive Status and Metric × Cognitive Status × Region interactions) were no longer significant after controlling for ADNI site supports the notion that differences in blood flow between MCI and CU older adults are small and variable across samples and may be an artifact of the relationship between regional and reference region CBF seen when using traditional rCBF (e.g., global hyper-perfusion in one group would present as higher CBF in the reference region and yield larger traditional rCBF values).

**Controlling for Hippocampal Volume**

Group differences in hippocampal rCBF were assessed with a Metric (traditional, residual) × Sex (male, female) × Cognitive Status (CU, MCI) mixed factorial ANCOVA analysis of covariance (ANCOVA) that controlled for age and hippocampal volume. Normalized bilateral hippocampal volume was calculated using a residual method in which the slope of the relationship between volume in the hippocampus and total intracranial volume was calculated within the CU group to estimate this metric in the absence of MCI-related atrophy (Jack et al., 1989). Eighteen participants were excluded from this analysis for missing intracranial volume estimates.

Results revealed a significant effect of Sex, *F*(1, 136) = 6.2, *p* = 0.014, η^2^ = 0.04, with higher rCBF in males than females. Significant effects of Metric, *F*(1, 136) = 151.6, *p* < 0.001, η^2^ = 0.53, and Metric × Sex, *F*(1, 136) = 7.5, *p* = 0.007, η^2^ = 0.05, revealed that the sex effects was larger for the residual (M_diff_: 10.36 ± 3.23), *p* = 0.002, than traditional (M_diff_: 6.67± 3.74), *p* = 0.077, rCBF metric.

**Controlling for APOE**

Group differences in rCBF were assessed with a Metric (traditional, residual) × Sex (male, female) × Cognitive Status (CU, MCI) × Region (frontal, parietal, temporal, occipital, hippocampal) mixed factorial ANCOVA analysis of covariance (ANCOVA) that controlled for age and apolipoprotein gene epsilon 4 (APOE ε4) status. Sixty participants were excluded from this analysis for missing APOE data.

Effects of Sex and Sex × Region were not significant, *p*s > 0.27. Significant effects of Metric, *F*(1, 94) = 1841.1, *p* < 0.001, η^2^ = 0.95, Metric × Sex, *F*(1, 94) = 5.2, *p* = 0.025, η^2^ = 0.05, and Metric × Sex × Region, *F*(4, 376) = 5.2, *p* < 0.001, η^2^ = 0.05, revealed a non-significant trend for lower rCBF in males than females in the occipital lobe that was larger for the traditional (M_diff_: -5.68 ± 3.57), *p* = 0.115, than residual (M_diff_: 4.18 ± 3.58), *p* = 0.246, rCBF metric, and a non-significant trend for higher rCBF in males than females in the hippocampus was larger for the residual (M_diff_: -6.58 ± 4.36), *p* = 0.135, than traditional (M_diff_: 2.61 ± 4.94), *p* = 0.598, rCBF metric. No other Sex group differences attained significance for other combinations of Metric or Region, *p*s > 0.25.

Significant effects of Metric × Cognitive Status, *F*(1, 94) = 5.8, *p* = 0.018, η^2^ = 0.06, and Metric × Cognitive Status × Region, *F*(4, 376) = 5.8, *p* < 0.001, η^2^ = 0.06, showed a non-significant trend for higher traditional rCBF in the MCI than CU group in the frontal lobe (M_diff_: 2.30 ± 1.50), *p* = 0.127 and a non-significant trend for lower traditional rCBF in the MCI than CU group in the hippocampus (M_diff_: -8.52 ± 4.98), *p* = 0.090. No other Cognitive Status group difference attained significance for other combinations of Metric or Region, *p*s > 0.16.

No interactions with Sex and Cognitive Status attained significance, *p*s > 0.28.
